# Overexpression of *PYL5* in rice enhances drought tolerance, inhibits growth, and modulates gene expression

**DOI:** 10.1093/jxb/ert397

**Published:** 2014-01-27

**Authors:** Hyunmi Kim, Kyeyoon Lee, Hyunsik Hwang, Nikita Bhatnagar, Dool-Yi Kim, In Sun Yoon, Myung-Ok Byun, Sun Tae Kim, Ki-Hong Jung, Beom-Gi Kim

**Affiliations:** ^1^Molecular Breeding Division, National Academy of Agricultural Science, RDA, Suwon 441-707, Republic of Korea; ^2^Department of Plant Molecular Systems Biotechnology and Crop Biotech Institute, Kyung Hee University, Yongin 446-701, Republic of Korea; ^3^Graduate School of Biotechnology, Kyung Hee University, Yongin 446-701, Republic of Korea; ^4^Department of Plant Bioscience, Pusan National University, Miryang, 627-706, Republic of Korea

**Keywords:** ABA receptors, drought stress, rice, salt stress.

## Abstract

Abscisic acid (ABA) is a phytohormone that plays important roles in the regulation of seed dormancy and adaptation to abiotic stresses. Previous work identified OsPYL/RCARs as functional ABA receptors regulating ABA-dependent gene expression in *Oryza sativa*. *OsPYL*/*RCAR*s thus are considered to be good candidate genes for improvement of abiotic stress tolerance in crops. This work demonstrates that the cytosolic ABA receptor OsPYL/RCAR5 in *O. sativa* functions as a positive regulator of abiotic stress-responsive gene expression. The constitutive expression of *OsPYL/RCAR5* in rice driven by the *Zea mays ubiquitin* promoter induced the expression of many stress-responsive genes even under normal growth conditions and resulted in improved drought and salt stress tolerance in rice. However, it slightly reduced plant height under paddy field conditions and severely reduced total seed yield. This suggests that, although exogenous expression of OsPYL/RCAR5 is able to improve abiotic stress tolerance in rice, fine regulation of its expression will be required to avoid deleterious effects on agricultural traits.

## Introduction

Plants have developed a multitude of mechanisms to survive under adverse conditions. Among these, plants can induce the expression of proteins that protect them from abiotic stresses, such as salt or drought. Several factors are involved in this transcriptional regulation (Shinozaki and Yamaguchi-Shinozaki, 2007; Cramer *et al.*, 2011; Santos *et al.*, 2011; Friedel *et al.*, 2012), and abscisic acid (ABA), in particular, plays pivotal roles in gene expression regulation for abiotic stress adaptation (Busk and Pages, 1998). ABA is synthesized or converted from an inactive form to an active form under stress conditions (Lee *et al.*, 2006; Xu *et al.*, 2012). The resulting increase in ABA concentration induces stress-responsive gene expression through ABA-signalling networks, which are well characterized in *Arabidopsis thaliana* and rice (Cutler *et al.*, 2010; Kim *et al.*, 2012). This ABA signalling includes components such as cytosolic ABA receptors PYRABACTIN RESISTANCE 1 LIKE/REGULATORY COMPONENTS OF ABA RECEPTORS (PYL/RCARs), clade A protein phosphatase 2Cs (PP2Cs), SNF1-related protein kinases 2 (SnRK2s), and basic leucine zipper (bZIP) transcription factors in *Arabidopsis* (Cutler *et al.*, 2010; Umezawa *et al.*, 2010).

These ABA-signalling components are highly conserved in land plants, in which drought tolerance is essential for survival. However, they are absent in the green algae *Chlamydomonas*, which lives in aquatic environments. This suggests that ABA signalling might have evolved due to the necessity for drought tolerance in land plants (Umezawa *et al.*, 2010). Compared with *Arabidopsis*, signalling components in the model monocot rice are similar in type and number (Hanada *et al.*, 2011; Kim *et al.*, 2012). Thus, ABA signalling appears to be functionally well conserved among *Arabidopsis*, rice, and other plants (Hanada *et al.*, 2011; Li *et al.*, 2011; Kim *et al.*, 2012; Wang *et al.*, 2012).

Previous work identified OsPYL/RCAR5, an orthologue of AtPYL/RCAR, as a functional ABA receptor in rice that interacts with OsPP2C9, OsPP2C49, and OsPP2C30, which are *Arabidopsis* subclass A PP2C orthologues. These interactions inactivate the PP2Cs, thereby releasing stress/ABA-activated protein kinases 2 (SAPK2) from suppression. Subsequently, SAPK2 activates OREB1, which induces the transcription of stress-responsive genes such as *RAB16A* and *LEA3* (Kim *et al.*, 2012).

In *Arabidopsis*, overexpression of AtPYL/RCAR5 causes not only ABA hypersensitivity but also drought stress tolerance. The transgenic plants show induction of stress-responsive genes and reduced water loss (Santiago *et al.*, 2009). Transgenic *Arabidopsis* plants constitutively overexpressing active PYL4 show enhanced drought tolerance compared with the wild-type PYL4 (Pizzio *et al.*, 2013). This suggested that rice ABA receptors might be a good candidate to improve drought tolerance in rice. The current work constructed transgenic rice constitutively overexpressing *OsPYL/RCAR5* (OsPYL/RCAR5-OE) and analysed the resulting abiotic stress-tolerance phenotype and genome-wide gene expression patterns comparing the OsPYL/RCAR5-OE line to the wild-type control. This study provides useful information for strategies aimed at improving abiotic stress tolerance and promotes the understanding of stress-responsive signalling network(s) downstream of the ABA receptor.

## Materials and methods

### Plasmid construction, rice transformation, and GUS assay

The rice cultivar used in this study was *Oryza sativa* cv. Dongjin. Dehulled rice seeds were surface-sterilized with 70% ethanol and 50% Clorox (Yuhanclorox, Korea) containing Tween 20 and washed with distilled water. *OsPYL/RCAR5* full-length cDNA was cloned into the pGA2897 vector including the maize *ubiquitin* promoter. For promoter analysis, promoters of *OsPYL/RCAR5* in entry vectors were introduced into the plant *GUS* reporter expression vector pBGWSF7 (Karimi *et al.*, 2002). The constructs were introduced into *Agrobacterium tumefaciens* LBA 4404 via electroporation. Transgenic rice was generated using the modified early scutellum *Agrobacterium*-mediated transformation method (Han *et al.*, 2012). The overexpression of transgenic rice was confirmed by reverse-transcription PCR (RT-PCR) and quantitative real-time PCR (qRT-PCR) as described previously (Kim *et al.*, 2012).

For GUS assays, T1 plants were grown on half-strength Murashige and Skoog (MS) medium (supplemented with 1% sucrose and 0.4% phytagel, adjusted to pH 5.8) for young seedling staining. GUS histochemical staining was performed using the substrate X-Glu as previously described (Lagarde *et al.*, 1996). GUS-stained plants and tissues were fixed by washing several times with 70% ethanol until the chlorophyll was completely removed from the tissue.

### Plant materials, growth conditions, and analysis of agricultural traits

Seeds of transgenic rice were germinated and grown for 1 week on 1/2 MS media containing hygromycin B (40mg l^–1^, Duchefa, Haarlem, Netherland) to select transgenic plants. After acclimation for 2 d in the greenhouse, the young seedlings were transferred to pots (16×6 × 5.5cm) filled with nursery soil and grown at 24– 30 °C for 4 weeks in the greenhouse. Those plants were planted in a paddy field in the middle of May, and seeds were harvested at the end of October annually for 2 years in 2011 and 2012 at the National Academy of Agricultural Science, Suwon, Korea. The paddy field soil is a sandy loam consisting of 0.7% gravel, 1.2% very coarse sand, 5.8% coarse sand, 18.4% medium sand, 23.9% find sand, 12.9% very fine sand, 29.1% silt, and 8.0 % clay. The pH of the soil was pH 6.0, and the soil contained (kg^–1^) 4.4 cmol extractable Ca^2+^, 0.27 cmol extractable K^+^, 1.4 cmol extractable Mg^2+^, 94mg available P_2_O_5_, and 24.5g total organic material. The water of paddy field was maintained 5cm above the soil from May to the end of September and then the water was drained and the watering was stopped. Nitrogen/phosphorus/potassium fertilizer (70:40:70kg ha^–1^) was applied after ploughing and 45 d after transplantation. The minimum and maximum temperatures and relative humidities recorded in a weather station during field cultivation from 2011 to 2012 are provided in Supplementary Fig. S1 (available at *JXB* online). To evaluate the agricultural traits of the transgenic rice, total grain weight, stem height, panicle length, number of panicles, and internode length were measured in 10 plants from each of three independent lines.

### Abiotic stress-tolerance assay

For the post-germination assay, surface-sterilized dehulled seeds were planted on 1/2 MS medium supplemented with hygromycin B (40mg l^–1^, Duchefa). Five days after planting, plants were transferred to 1/2 MS media supplemented with 200 or 400mM mannitol, or 5 µM ABA in square Petri dishes (125×125×20mm) and grown vertically under long-day conditions (16/8 light/dark cycle) in a growth incubator (model FLI-2000, Eyela, Tokyo, Japan). Seedling growth was then measured 7 d later.

Preliminary drought-tolerance assays used 18 T2 seeds and was performed in a greenhouse where temperature was ranged from 20 to 35 °C and natural sunlight was used as illumination. Three-week-old young transgenic rice and control plants were grown in the same pot containing nursery soil for 3 weeks and watering was stopped for 5–7 d (depending on the weather) until leaves wilted, at which point the plants were rewatered.

For further drought-tolerance assays, germinated seeds were transferred to pots containing nursery soil with 41.6±0.5 % water content and grown for 3 weeks. Watering was stopped for 5 d when gravimetric soil water content was 7.2±0.85%, when the plants were rewatered. The freshweight of the plants was measured.

For the salt stress assay, germinated seeds were transferred into pots containing nursery soil and grown for 3 weeks. The pots were transferred to a container filled with 200mM NaCl up to 2cm from the bottom and were then incubated for 8 or 9 d, with the NaCl solution being supplemented twice to maintain the water depth. Pictures were taken and the freshweight of the plants was measured. For the water loss assay, germinated seeds were transferred into pots containing nursery soil and grown for 3 weeks. Three flag leaves per independent line were cut and kept at 28 °C on filter paper under 150 μmol m^–2^ s^–1^ in the tissue culture room. Weighing was performed every 10 minutes for 3h.

For the whole-plant transpiration assay, germinated seeds were transferred into soil-filled 50-ml tubes and grown until the third leaf was observed. Before commencing the whole-plant transpiration assay, plants were watered and tubes were sealed with wrap to prevent soil water evaporation. The transpiration rate was monitored every 12h by weighing the tube until leaves were wilted. To measure the leaf surface area, cut leaves were placed in water to rehydrate until turgid, then leaf surface area was measured using a LI-3000A portable area meter (LI-COR, USA).

### Microarray analysis

For microarray analysis, 2-week-old Dong-jin and OsPYL/RCAR5-OE rice seedlings were grown in MS media. For biological replicates, seedlings were sampled independently three times. Total RNA was extracted using a Mini RNA kit (Qiagen) and analysed using Rice Gene Expression Microarray and Gene Expression Hybridization kits (Agilent), according to the manufacturer’s instructions. The signals were scanned using an Agilent DNA microarray scanner and signal intensity for individual probes was analysed using Agilent Feature Extraction Software version 7.5.1. The intensity was normalized by the quantile method and translated into the log_2_ scale (Bolstad *et al.*, 2003).

The log_2_-normalized intensity data was then uploaded from a tab-delimited text file format to Multi Experiment Viewer (MEV, http://www.tm4.org/mev/; Saeed *et al.*, 2006) and a heat map was generated. In addition, the heat map image based on log_2_-fold-change data in response to drought stress was also created. For the drought stress analysis, this work compiled the microarray data from ArrayExpress (http://www.ebi.ac.uk/arrayexpress/) and from a public microarray database, NCBI GEO (http://www.ncbi.nlm.nih.gov/geo/, platform accession number GPL2025, data series accession numbers GSE6901, GSE24048, GSE26280, and E-MEXP-2401: 31 comparisons). Fold-change data under various drought stresses were then integrated with the fold-change of OsPYL/RCAR5-OE over Dong-jin (control plant). To identify significant expression patterns, genes that had greater or lesser than 2-fold-change under coefficient of variation of less than 1 were chosen.

### Gene ontology analysis

Gene ontology (GO) analysis was done for 61 genes commonly upregulated and 69 genes commonly downregulated in OsPYL/RCAR5-OE plants compared to the wild type, representing significant expression during drought stress. The MSU locus identifiers of genes were queried in the Rice Genome Annotation Project (http://rice.plantbiology.msu.edu/downloads_gad.shtml) for GO terms and annotations. The details that were retrieved from the database were then shortlisted for biological processes and ordered based on the number of genes assigned to a GO term, with only those presenting more than two genes being considered. Graphical representations were prepared to state the number of genes associated with various biological processes.

## Results

### Expression of *OsPYL/RCAR5* in different tissues and stress conditions

**Fig. 1.**
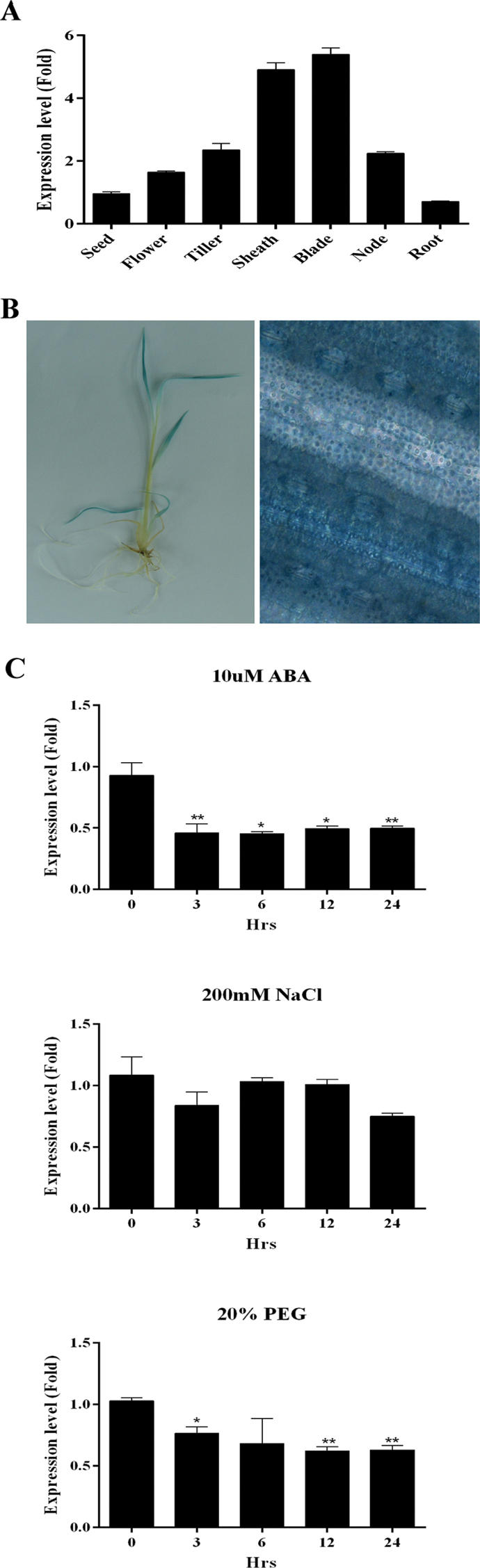
Expression pattern of *OsPYL/RCAR5* in different tissues and stress conditions. (A) qRT-PCR analysis of *OsPYL/RCAR5* mRNA levels in several tissues of mature wild-type rice plants; *Ubi5* was used as a reference gene. (B) Histochemical β-glucosidase analysis of *OsPYL/RCAR5* promoter-GUS transgenic rice; 10-d-old seedling (left) and microscopic analysis at ×400 magnification (right). (C) qRT-PCR analysis of *OsPYL/RCAR5* mRNA levels isolated in young seedlings treated with stress conditions for the indicated periods of time; ABA treatment (top), NaCl treatment (middle), and PEG 3300 treatment (bottom). Error bars represent standard error of three replicates. Asterisks above the bars indicate significant differences between lines: *P < 0.05, **P < 0.01.

*OsPYL*/*RCAR5* (*Os05g12260*) expression in different tissues was analysed by qRT-PCR. It was expressed in all tissues examined and was the most abundant in the leaf blade, followed by the leaf sheath, and was the lowest in root tissue (Fig. 1A). To confirm these results, transgenic plants harbouring an *OsPYL/RCAR5* promoter-GUS fusion cassette were also produced and analysed. Strong GUS staining was detected in the leaf blades of the young seedlings, but did not observe staining in the roots of young seedlings (Fig. 1B). Microscopic analysis of leaf blades stained using X-Glu showed that the *OsPYL/RCAR5* promoter drove GUS expression throughout the surface of the leaf blade, including guard cells (Fig. 1B). Taking these results together, it was concluded that *OsPYL/RCAR5* is expressed abundantly in aerial parts such as the leaf blade and leaf sheath. However, *OsPYL/RCAR5* was expressed at very low levels in roots and seeds compared to leaves.

In order to identify the gene expression pattern in response to several abiotic stressors, qRT-PCR analysis was performed using young rice seedlings treated with ABA, PEG (osmotic stress), or salt stress (Fig. 1C). Interestingly, the expression of *OsPYL/RCAR5* was repressed under ABA or PEG treatment and was changed marginally under NaCl treatment. Thus, *OsPYL/RCAR5* is not induced and is actually slightly suppressed by certain abiotic stresses.

### Production of transgenic rice overexpressing *OsPYL/RCAR5*

**Fig. 2.**
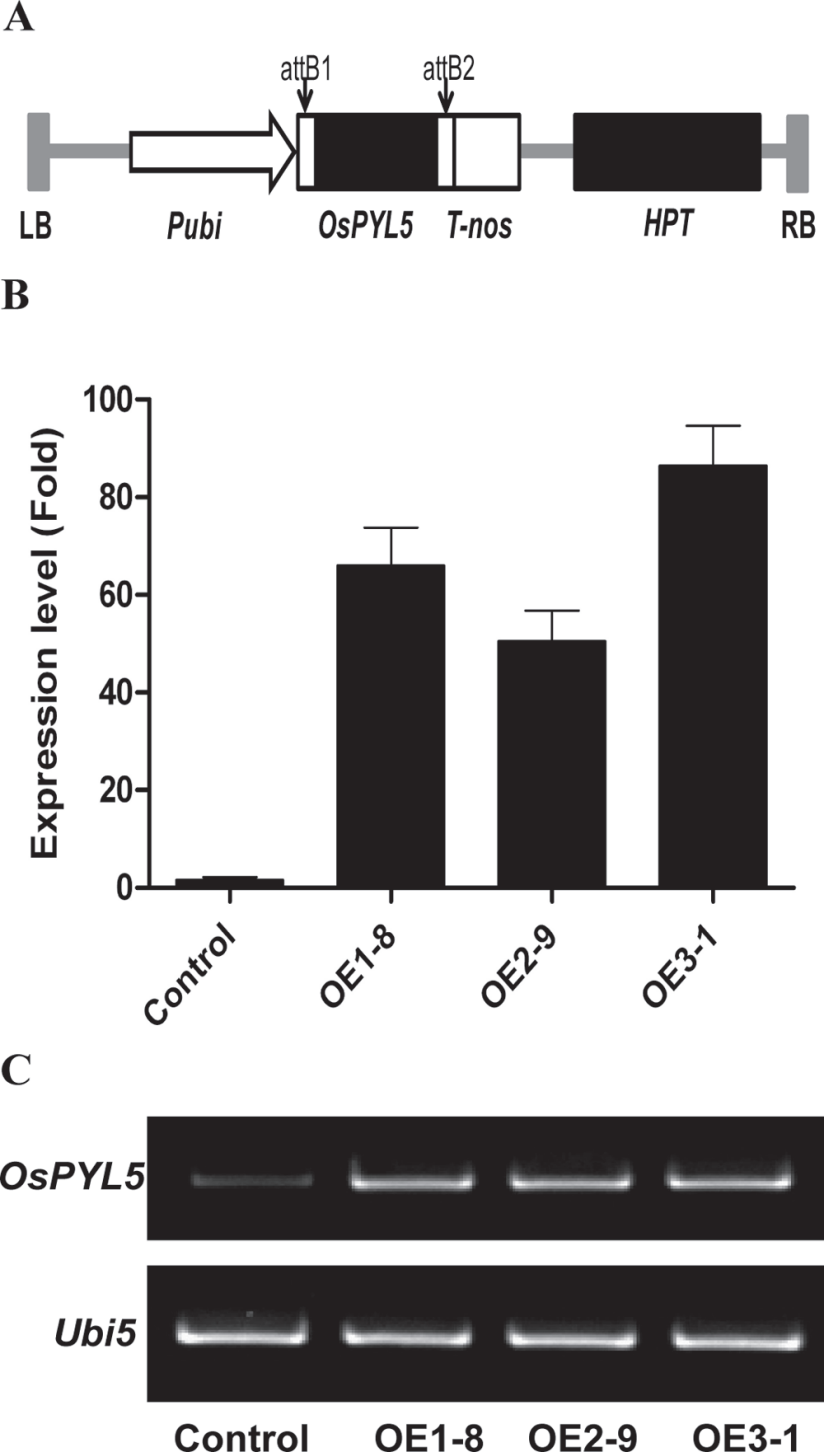
Construction of transgenic rice overexpressing *OsPYL/RCAR5*. (A) Schematic diagram of the pUbi-*OsPYL/RCAR5* construct used for transformation; LB, left border; Pubi, maize *ubiquitin* promoter; *OsPYL/RCAR5*, OsPYL/RCAR5 cDNA; T-nos, nos terminator; HPT, hygromycin B phosphotransferase; RB, right border. (B and C) qRT-PCR (B) and RT-PCR (C) analysis of *OsPYL/RCAR5* expression in the overexpression lines and control (transgenic rice containing the empty pGA2897 vector). OE1-8, OE2-9, and OE3-1 are three individual transgenic plants overexpressing *OsPYL/RCAR5* gene.

To construct transgenic rice overexpressing *OsPYL/RCAR5*, cDNA was fused with the maize *ubiquitin* promoter in the pGA2897 vector (Fig. 2A). The construct was transformed into *O. sativa* L. cv. Dong-jin, producing a total of 20 independent transgenic rice lines. The insertion and overexpression of *OsPYL/RCAR5* was confirmed by genomic DNA PCR and RT-PCR analyses using T0 plants (data not shown). Because many of the plants were very short and produced very few seeds compared to control plants, six T0 lines that had the heaviest total grain weight, were the tallest, and/or exhibited high *PYL5* overexpression were selected for further analysis. Five plants per independent T1 line were grown in the paddy field and 30 T2 lines in total were harvested. Three lines were selected among five T2 lines originated from each independent T0 line. From drought-tolerance assays using 18 T2 seeds, three T2 transgenic plants that exhibited the strongest drought stress tolerance in preliminary experiments were chosen for further analyses (OsPYL/RCAR5-OE lines 1–3). First, the expression of *OsPYL/RCAR5* in these lines was confirmed by qRT-PCR (Fig. 2B). All three independent lines displayed *OsPYL/RCAR5* expression levels at least 50-fold higher than the control line. These results were also confirmed by agarose gel electrophoresis of RT-PCR products (Fig. 2C).

### Ectopic expression of *OsPYL/RCAR5* in transgenic rice leads to drought and salt tolerance in the vegetative growth stage

**Fig. 3.**
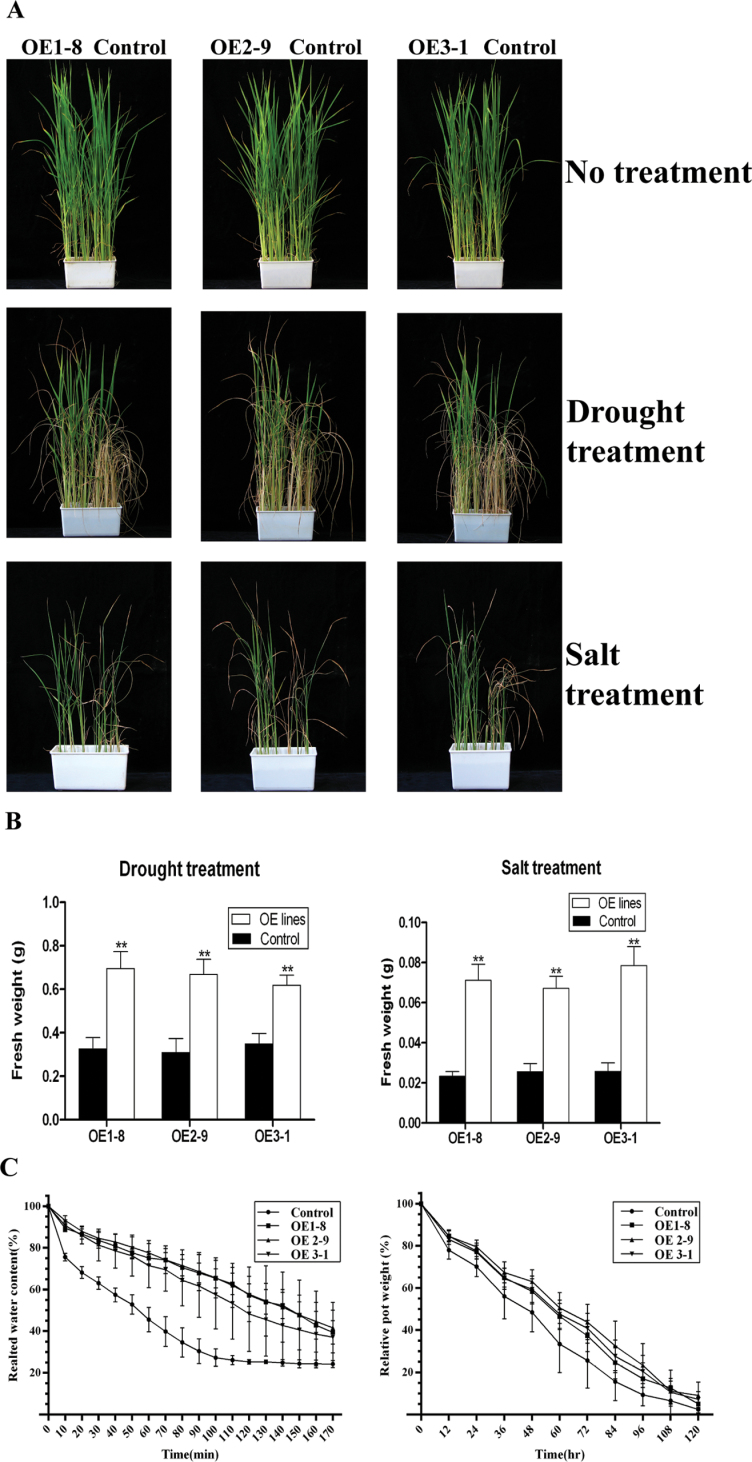
Transgenic rice overexpressing *OsPYL/RCAR5* exhibits enhanced drought and salt tolerance at the vegetative stage. (A) Three-week-old plants were watered with tap water supplemented with 200mM NaCl or rewatered after 5 d of drought treatment; the experiments were repeated three times, with consistent results; representative images and graphs are shown. (B) Freshweight of transgenic plants grown under abiotic stress conditions; error bars represent standard error of 12 replicates; asterisks above the bars indicate significant differences between lines: **P < 0.01. (C) Water loss assay (left) and whole-plant transpiration assay (right); error bars represent standard error of three and five replicates, respectively.

To identify whether ectopic expression of *OsPYL/RCAR5* can confer abiotic stress tolerance to rice, seedlings were subjected to drought or salt stress in growth chambers. For the drought-tolerance assay, 3-week-old plants were rewatered after 5 d of drying. More leaves survived in the OsPYL/RCAR5-OE plants than in control plants (Fig. 3A), and the freshweights of the OsPYL/RCAR5-OE plants were almost 2-fold higher than those of control plants (Fig. 3B). The three independent transgenic lines showed similar phenotypes. These results indicate that overexpression of the ABA receptor *OsPYL/RCAR5* can increase drought tolerance in rice at the vegetative stage.

To determine whether stomatal closure is involved in the drought tolerance of OsPYL/RCAR5-OE transgenic plants, detached-leaf and whole-plant transpiration assays were performed. In the detached-leaf transpiration assays, all transgenic plant leaves lost water slowly and reached equilibrium later than control. At 100min, control plant leaves reached equilibrium and had around 27% relative freshweight. However, OsPYL/RCAR5-OE transgenic plant leaves retained about 60% relative freshweight at the same time point (Fig. 3C).

In the whole-plant transpiration assay, OsPYL/RCAR5-OE showed a transpiration rate of about 10% less than control plants during drought stress treatment (Fig. 3C). These two different assays both indicated that the drought tolerance of OsPYL/RCAR5-OE transgenic plants was related to enhanced stomatal closure.

For the salt-tolerance assay, 3-week-old plants were incubated with 200mM NaCl for 8 or 9 d. Similar to the results for drought tolerance, more leaves survived in the OsPYL/RCAR5-OE transgenic plants than in control plants under salt stress conditions (Fig. 3A). The freshweights of OsPYL/RCAR5-OE plants were also 3-fold heavier than those of control plants under salt stress (Fig. 3B).

Taken together, these results demonstrate that *OsPYL/RCAR5* overexpression can enhance drought and salt tolerance at the vegetative growth stage.

### Growth of transgenic plants overexpressing *OsPYL/RCAR5* is retarded under osmotic stress and ABA treatment

**Fig. 4.**
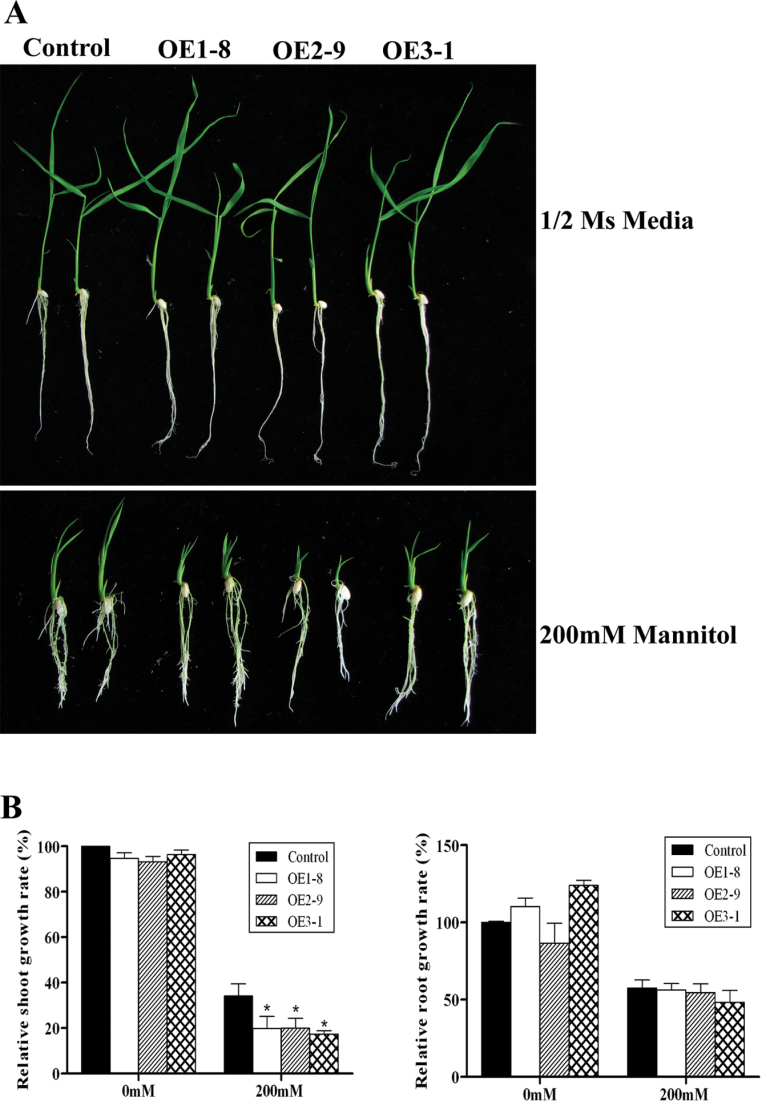
*OsPYL/RCAR5*-overexpressing transgenic rice exhibits growth inhibition under osmotic stress conditions. (A) Seedling growth in post-germination assays on 1/2 MS plates without or with 200mM mannitol. (B) Relative shoot growth (left) and relative root growth (right); error bars represent standard error of 10 replicates: *P < 0.05 (this figure is available in colour at *JXB* online).

**Fig. 5.**
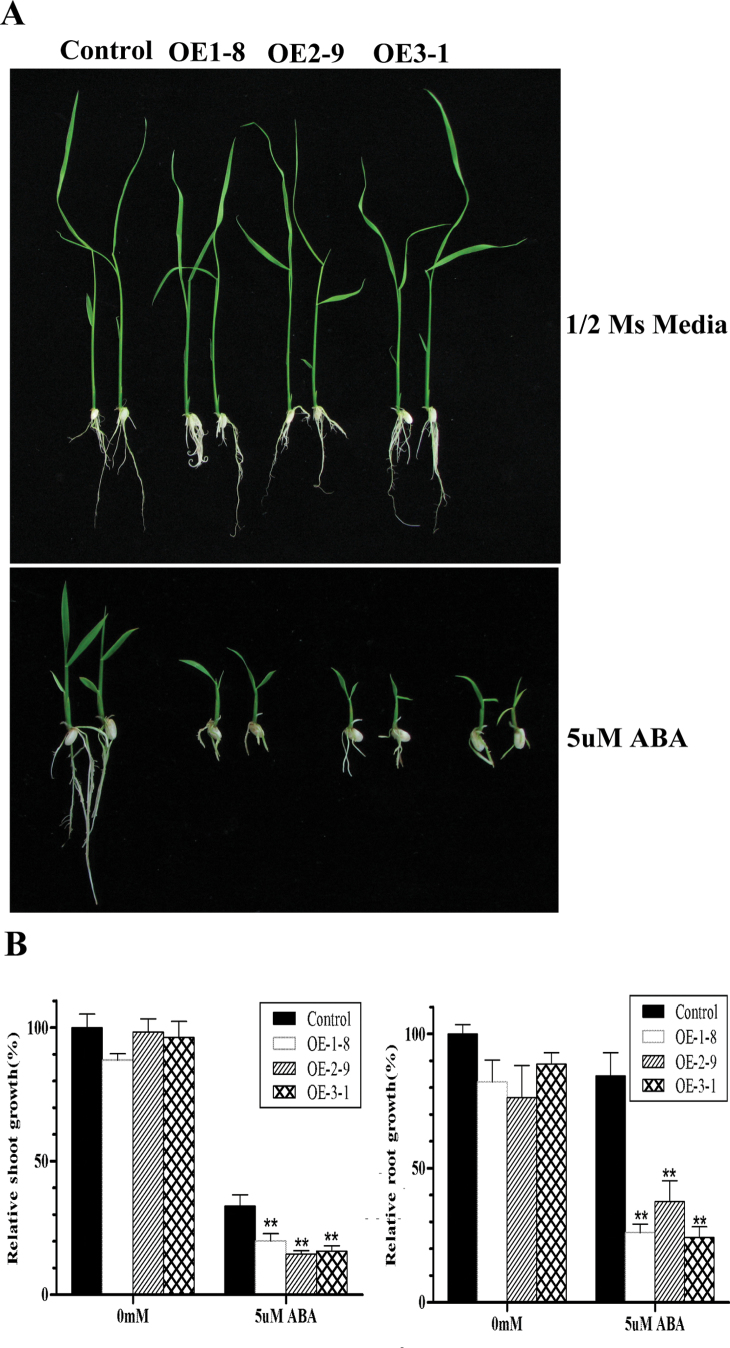
*OsPYL/RCAR5*-overexpressing transgenic rice exhibits hypersensitivity to ABA. (A) Seedling growth in post-germination assays on 1/2 MS plates without or with 5 μM ABA. (B) Relative shoot growth (left) and relative root growth (right); error bars represent standard error of five replicates: *P < 0.05 (this figure is available in colour at *JXB* online).

To extend the osmotic stress-tolerance analysis, post-germination assays were performed using plants grown on plates. No significant differences were observed in the growth of transgenic rice on 1/2 MS medium without other treatments. Three days after germination on 1/2 MS medium, the seedlings were transferred to 1/2 MS medium containing different concentrations of mannitol or NaCl, and the shoot and root lengths were measured after 7 d. There were no significant differences in root growth between control and OsPYL/RCAR5-OE lines (Fig. 4A). By contrast, in the presence of 200mM mannitol, the shoot growth of OsPYL/RCAR5-OE plants was inhibited more than 10% compared to that of the control (Fig. 4B). The assays showed an ABA-hypersensitive phenotype in the transgenic rice lines. The growth of both roots and shoots of the OsPYL/RCAR5-OE plants was inhibited more than 50% in the 5 μM ABA condition compared to growth in MS media (Fig. 5).

### Transgenic plants overexpressing *OsPYL/RCAR5* have dwarf phenotypes and show reduced yield

**Fig. 6.**
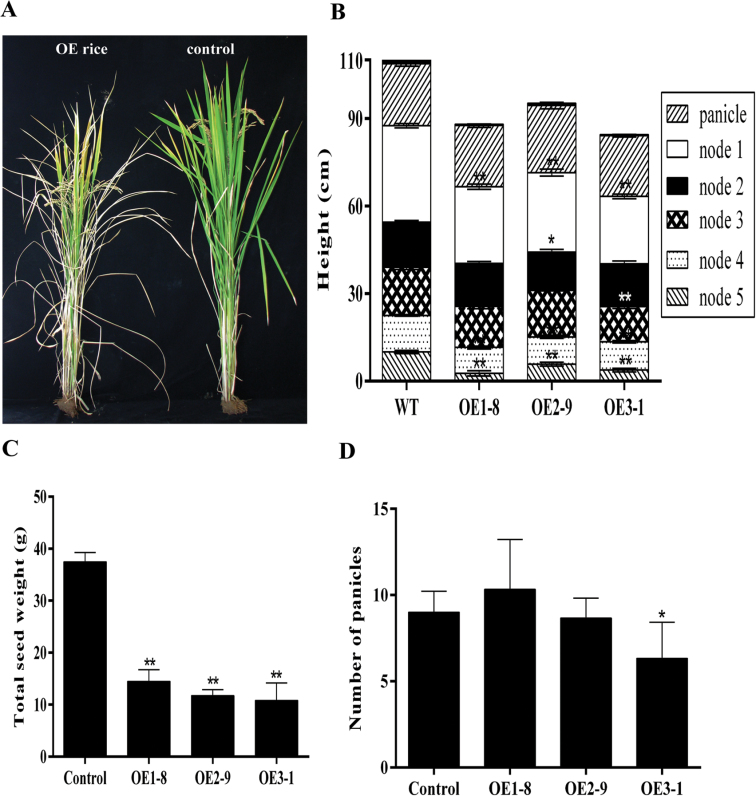
Agricultural traits of rice overexpressing *OsPYL/RCAR5*. (A) Morphology of mature rice grown in a paddy field. (B) Stem heights showing each internode length. (C) Total seed weight. (D) Number of panicles per plant. Error bars represent standard error of eight replicates. Asterisks above the bars indicate significant differences between lines: *P < 0.05, **P < 0.01.

To examine the agricultural traits of the OsPYL/RCAR5-OE lines, they were grown in paddy fields and four agronomic traits were examined. Mature OsPYL/RCAR5-OE lines showed semi-dwarfism, even though severe differences were not observed until the four-leaf stage (Fig. 6A and B). Overall, the stems of mature OsPYL/RCAR5-OE plants were around 10% shorter than those of the wild type. When the lengths of the individual internodes were compared, each internode was slightly shorter than that of the control plants (Fig. 6B), revealing that growth inhibition occurred in all internodes and that there were no specific differential effects on particular internodes (Fig. 6B). Despite the reduced height, there was no significant difference in tiller number between transgenic and control plants (Fig. 6D). However, the total grain weight of the OsPYL/RCAR5-OE lines was approximately one-third that of control plants (Fig. 6C).

### Expression of abiotic stress-responsive genes is altered in transgenic rice overexpressing *OsPYL/RCAR5*

**Fig. 7.**
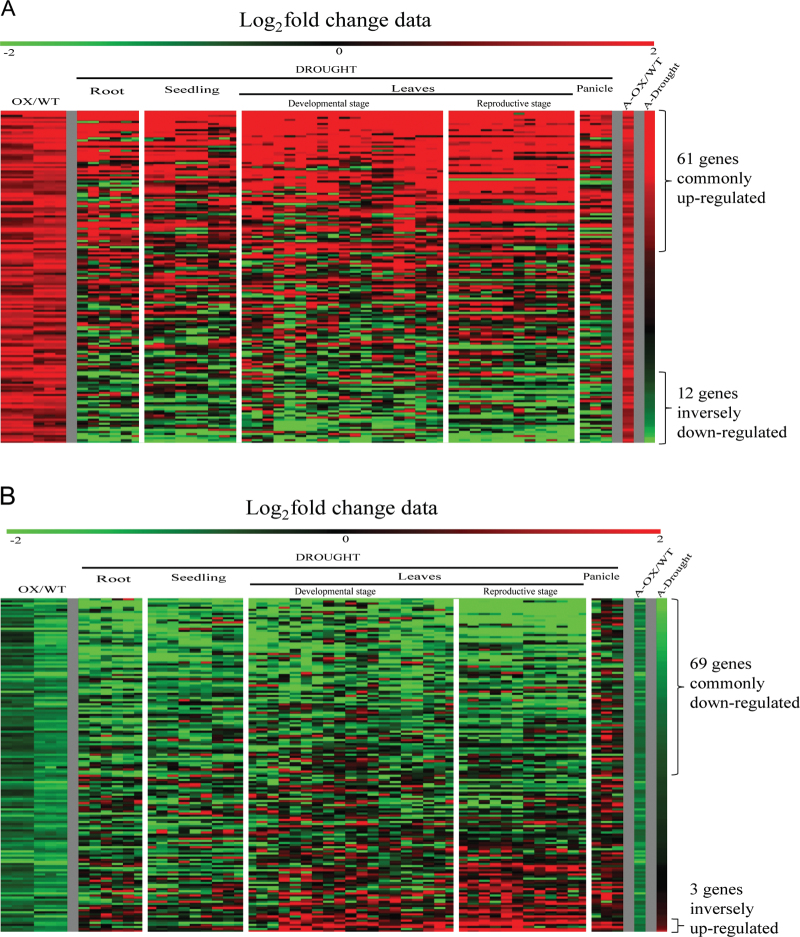
Heat map of expression patterns under various drought stresses for genes differentially expressed in OsPYL/RCAR5-OE plants compared with control plants: genes up-regulated (A) and down-regulated (B) in OsPYL/RCAR5-OE plants. OX/WT indicates log_2_ fold change of OsPYL/RCAR5-OE plant over wild type plant. Log_2_ fold change data indicates average log_2_ fold change value of 31 drought stress treatments compared to untreated control from public database.

Since OsPYL/RCARs plays pivotal roles in ABA-dependent transcriptional regulation, this work performed genome-wide microarray analysis of OsPYL/RCAR5-OE and compared the results with transcriptomic data under drought stress that was downloaded from ArrayExpress and from the public microarray database NCBI GEO. A total of 162 genes 2-fold upregulated and 159 genes 2-fold downregulated were identified in OsPYL/RCAR5-OE compared with the wild type under normal growth condition (Supplementary Table S1). Of the former, 61 genes were commonly upregulated by both drought and overexpression of *OsPYL/RCAR5*, and 12 genes showed opposite differential expression with a change more than 2-fold (Fig. 7A). Of the latter, 69 genes were commonly downregulated and three genes inversely upregulated with respect to drought (Supplementary Table S2 and Fig. 7B). These analyses show that many genes induced by drought treatment are already expressed in OsPYL/RCAR5-OE plants even under normal growth conditions. The other genes differentially expressed in wild-type and OsPYL/RCAR5-OE plants might explain the growth retardation phenotype of OsPYL/RCAR5-OE plants.

### Gene ontology analysis for functional classification

**Fig. 8.**
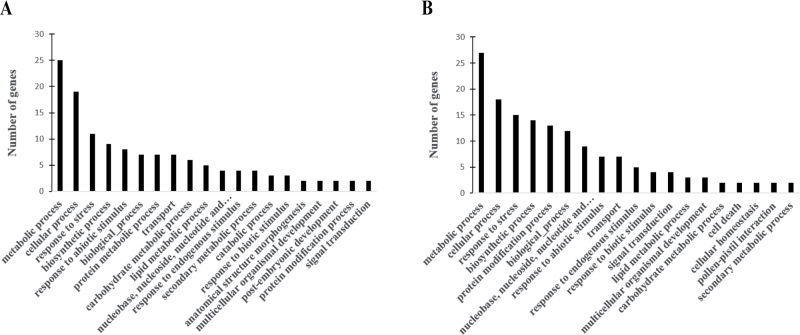
GO analysis in the biological process category for genes differentially expressed in OsPYL/RCAR5-OE and control plants and under drought and control treatments: genes upregulated (A) and downregulated (B) in OsPYL/RCAR5-OE plants.

To discern the biological meaning of genes commonly differentially expressed by drought and in OsPYL/RCAR5-OE plants, GO annotation analysis in the biological process category was carried out for 61 genes commonly upregulated and 69 genes commonly downregulated in OsPYL/RCAR5-OE plants compared to the wild type and drought compared to control treatment. Among the commonly upregulated genes, the majority were associated with metabolic processes, cellular processes, and response to stress (Fig. 8A), and similar results were obtained for commonly downregulated genes (Fig. 8B).

## Discussion

With the discovery of PYL/RCARs as ABA receptors, the last piece of the puzzle of signalling networks for ABRE-mediated ABA-dependent gene expression was revealed (Ma *et al.*, 2009; Park *et al.*, 2009). These signalling networks have been recently reported in other plants, including rice, beechnut, and cucumber (Saavedra *et al.*, 2010; Kim *et al.*, 2012; Romero *et al.*, 2012; Wang *et al.*, 2012). Genes involved in ABA signalling are prime targets to improve abiotic stress tolerance of plants. AtPYL/RCAR5 enhances stress tolerance when constitutively overexpressed in *Arabidopsis*. Moreover, a constitutively activated (CA) PYR1 ABA receptor that interacts with PP2C without ABA was engineered successfully using site-saturated mutagenesis (Mosquna *et al.*, 2011). Also, *Arabidopsis* lines constitutively overexpressing CA PYL4 showed enhanced drought tolerance compared to *Arabidopsis* lines overexpressing wild-type PYL4 (Pizzio *et al.*, 2013). These results suggest that ABA receptors are candidates to improve abiotic stress tolerance of crops. However, it has not yet reported whether the overexpression of ABA receptors in a monocotyledonous species can enhance abiotic stress tolerance. This study thus provides the first report in a monocot model plant (rice) that constitutive overexpression of a cytosolic ABA receptor (*OsPYL/RCAR5*) confers not only drought tolerance but also salt tolerance.

Abiotic stress-tolerant or ABA-hypersensitive plants might show growth retardation because of hypersensitivity in terms of recognizing and responding to stresses, directing resources to protection from stresses rather than growing or yielding. Thus it is a general problem that genes improving abiotic stress tolerance cause growth retardation even under normal growth conditions when they are constitutively overexpressed. In particular, constitutive overexpression of ABA-signalling-related genes might significantly retard growth, since ABA plays a pivotal role in plant development and growth regulation (Sreenivasulu *et al.*, 2012). For example, DREB1A-overexpressing plants driven by the 35S cauliflower mosaic virus promoter showed reduced growth under normal conditions (Kasuga *et al.*, 1999; Nakashima *et al.*, 2007). Transgenic plants overexpressing the *Arabidopsis* downregulating β-subunit of farnesyltransferase (AtFTB), a negative regulator of ABA signalling, are drought tolerant but also show seedling growth inhibition (Wang *et al.*, 2005). To solve this problem, stress-inducible promoters such as rd29A have been used and have successfully solved the problem to some degree (Kasuga *et al.*, 1999; Wang *et al.*, 2005).

Young OsPYL/RCAR5-OE seedlings, when grown under optimized conditions in growth chambers or on plates, also showed slight growth retardation. However, when grown to maturity in paddy fields, OsPYL/RCAR5-OE plants showed severe growth retardation, in terms of internode length and leaf length and severely reduced total seed yields. In the paddy field, there are many stress factors which cannot be regulated, such as cold, heat, drought, and high light. For example, based on the minimum field temperatures (Supplementary Fig. S1), 25 and 19 d were recorded with a minimum temperature under 17 °C from May 21 to end of September in 2011 and 2012, respectively. Temperatures under 17 °C might have given mild cold stress to rice vegetative growth and pollination (Suh *et al.*, 2010). If OsPYL/RCAR5-OE responds excessively to mild cold stress, this could explain the retarded growth of OsPYL/RCAR5-OE plants compared to control plants, at least in part.

The overproduced OsPYL/RCAR5 proteins might sequester subclass A PP2Cs and keep them inactive through interaction in normal conditions with low levels of ABA. Thus, the SAPKs could be in an active state in normal or in mild stress conditions because subclass A PP2C cannot inhibit SAPK activity (Cutler *et al.*, 2010; Umezawa *et al.*, 2010). This could underlie the excessive responsiveness of OsPYL/RCAR5-OE rice lines to abiotic stresses. In addition, OsPYL/RCAR5-OE rice could be in a stress-responsive state even under normal growth conditions.

Transcriptome and meta-expression analyses also support the idea that OsPYL/RCAR5-OE plants activate the stress-responsive gene expression signalling network even in the absence of stress treatment. Microarray analysis of OsPYL/RCAR5-OE vs. the wild type revealed that ~321 genes were induced or repressed at least 2-fold in transgenic rice even in the absence of osmotic stress. Of these, 61 genes upregulated in OsPYL/RCAR5-OE were also upregulated by drought treatments (Fig. 7). In particular, typical drought-stress-responsive genes such as LEA, dehydrin, and Hsp were induced in OsPYL/RCAR5-OE plants even in the absence of osmotic stress.

Taken together, these results demonstrate that *OsPYL/RCAR5*, encoding the first functional ABA receptor reported in monocots, is a good candidate gene for the improvement of abiotic stress tolerance in monocot crops, although further studies for fine regulation of gene expression are required to avoid negative effects on growth and grain yield.

## Supplementary material

Supplementary data are available at *JXB* online.

Supplementary Fig. S1. Seasonal variations of temperatures and relative humidities of the rice paddy field in the years 2011 and 2012.

Supplementary Table S1. List of 2-fold up- and downregulated genes in OsPYL/RCAR5-OE compared with wild type under normal growth conditions.

Supplementary Table S2. List of commonly up-, down-, or inversely regulated genes compared with drought-regulated genes.

Supplementary Data

## References

[CIT0001] Bolstad BM Irizarry RA Astrand M Speed TP 2003 A comparison of normalization methods for high density oligonucleotide array data based on variance and bias. Bioinformatics 19, 185–193 12538238 10.1093/bioinformatics/19.2.185

[CIT0002] Busk PK Pages M 1998 Regulation of abscisic acid-induced transcription. Plant Molecular Biology 37, 425–435 9617810 10.1023/a:1006058700720

[CIT0003] Cramer GR Urano K Delrot S Pezzotti M Shinozaki K 2011 Effects of abiotic stress on plants: a systems biology perspective. BMC Plant Biology 11, 163 22094046 10.1186/1471-2229-11-163 PMC3252258

[CIT0004] Cutler SR Rodriguez PL Finkelstein RR Abrams SR 2010 Abscisic acid: emergence of a core signaling network. Annual Review in Plant Biology 61, 651–679 10.1146/annurev-arplant-042809-112122 20192755

[CIT0005] Friedel S Usadel B von Wiren N Sreenivasulu N 2012 Reverse engineering: a key component of systems biology to unravel global abiotic stress cross-talk. Frontiers in Plant Science 3, 294 23293646 10.3389/fpls.2012.00294 PMC3533172

[CIT0006] Han S Shin D Moon S Jeon S Byun M Kim B 2012 Optimization of *Agrobacterium*-mediated transformation in japonica-type rice *Oryza sativa* L. cv. Dongjin for high efficiency. Korean Journal of Breeding Science 44, 221–228

[CIT0007] Hanada K Hase T Toyoda T Shinozaki K Okamoto M 2011 Origin and evolution of genes related to ABA metabolism and its signaling pathways. Journal of Plant Research 124, 455–465 21626211 10.1007/s10265-011-0431-0

[CIT0008] Kasuga M Liu Q Miura S Yamaguchi-Shinozaki K Shinozaki K 1999 Improving plant drought, salt, and freezing tolerance by gene transfer of a single stress-inducible transcription factor. Nature Biotechnology 17, 287–291 10.1038/7036 10096298

[CIT0009] Kim H Hwang H Hong JW Lee YN Ahn IP Yoon IS Yoo SD Lee S Lee SC Kim BG 2012 A rice orthologue of the ABA receptor, OsPYL/RCAR5, is a positive regulator of the ABA signal transduction pathway in seed germination and early seedling growth. Journal of Experimental Botony 63, 1013–1024 10.1093/jxb/err338 22071266

[CIT0010] Lagarde D Basset M Lepetit M Conejero G Gaymard F Astruc S Grignon C 1996 Tissue-specific expression of *Arabidopsis* AKT1 gene is consistent with a role in K+ nutrition. The Plant Journal 9, 195–203 8820606 10.1046/j.1365-313x.1996.09020195.x

[CIT0011] Lee KH Piao HL Kim HY Choi SM Jiang F Hartung W Hwang I Kwak JM Lee IJ 2006 Activation of glucosidase via stress-induced polymerization rapidly increases active pools of abscisic acid. Cell 126, 1109–1120 16990135 10.1016/j.cell.2006.07.034

[CIT0012] Li C Jia H Chai Y Shen Y 2011 Abscisic acid perception and signaling transduction in strawberry: a model for non-climacteric fruit ripening. Plant Signaling and Behavior 6, 1950–1953 22095148 10.4161/psb.6.12.18024 PMC3337185

[CIT0013] Ma Y Szostkiewicz I Korte A Moes D Yang Y Christmann A Grill E 2009 Regulators of PP2C phosphatase activity function as abscisic acid sensors. Science 324, 1064–1068 19407143 10.1126/science.1172408

[CIT0014] Mosquna A Peterson FC Park SY Lozano-Juste J Volkman BF Cutler SR 2011 Potent and selective activation of abscisic acid receptors in vivo by mutational stabilization of their agonist-bound conformation. Proceedings of the National Academy of Sciences, USA 108, 20838–20843 10.1073/pnas.1112838108 PMC3251050 22139369

[CIT0015] Nakashima K Tran LS Van Nguyen D Fujita M Maruyama K Todaka D Ito Y Hayashi N Shinozaki K Yamaguchi-Shinozaki K 2007 Functional analysis of a NAC-type transcription factor OsNAC6 involved in abiotic and biotic stress-responsive gene expression in rice. The Plant Journal 51, 617–630 17587305 10.1111/j.1365-313X.2007.03168.x

[CIT0016] Park SY Fung P Nishimura N 2009 Abscisic acid inhibits type 2C protein phosphatases via the PYR/PYL family of START proteins. Science 324, 1068–1071 19407142 10.1126/science.1173041 PMC2827199

[CIT0017] Pizzio GA Rodriguez L Antoni R Gonzalez-Guzman M Yunta C Merilo E Kollist H Albert A Rodriguez PL 2013 The PYL4 A194T mutant uncovers a key role of PYR1-LIKE4/PROTEIN PHOSPHATASE 2CA interaction for abscisic acid signaling and plant drought resistance. Plant Physiology 163, 441–455 23864556 10.1104/pp.113.224162 PMC3762663

[CIT0018] Romero P Lafuente MT Rodrigo MJ 2012 The Citrus ABA signalosome: identification and transcriptional regulation during sweet orange fruit ripening and leaf dehydration. Journal of Experimental Botony 63, 4931–4945 10.1093/jxb/ers168 PMC3428003 22888124

[CIT0019] Saavedra X Modrego A Rodriguez D Gonzalez-Garcia MP Sanz L Nicolas G Lorenzo O 2010 The nuclear interactor PYL8/RCAR3 of *Fagus sylvatica* FsPP2C1 is a positive regulator of abscisic acid signaling in seeds and stress. Plant Physiology 152, 133–150 19889877 10.1104/pp.109.146381 PMC2799352

[CIT0020] Saeed AI Bhagabati NK Braisted JC Liang W Sharov V Howe EA Li J Thiagarajan M White JA Quackenbush J 2006 TM4 microarray software suite. Methods in Enzymology 411, 134–193 16939790 10.1016/S0076-6879(06)11009-5

[CIT0021] Santiago J Rodrigues A Saez A Rubio S Antoni R Dupeux F Park SY Marquez JA Cutler SR Rodriguez PL 2009 Modulation of drought resistance by the abscisic acid receptor PYL5 through inhibition of clade A PP2Cs. The Plant Journal 60, 575–588 19624469 10.1111/j.1365-313X.2009.03981.x

[CIT0022] Santos AP Serra T Figueiredo DD Barros P Lourenco T Chander S Oliveira MM Saibo NJ 2011 Transcription regulation of abiotic stress responses in rice: a combined action of transcription factors and epigenetic mechanisms. OMICS 15, 839–857 22136664 10.1089/omi.2011.0095

[CIT0023] Shinozaki K Yamaguchi-Shinozaki K 2007 Gene networks involved in drought stress response and tolerance. Journal of Experimental Botony 58, 221–227 10.1093/jxb/erl164 17075077

[CIT0024] Sreenivasulu N Harshavardhan VT Govind G Seiler C Kohli A 2012 Contrapuntal role of ABA: does it mediate stress tolerance or plant growth retardation under long-term drought stress? Gene 506, 265–273 22771691 10.1016/j.gene.2012.06.076

[CIT0025] Suh JP Jeung JU Lee JI Choi YH Yea JD Virk PS Mackill DJ Jena KK 2010 Identification and analysis of QTLs controlling cold tolerance at the reproductive stage and validation of effective QTLs in cold-tolerant genotypes of rice (*Oryza sativa* L.). Theoretical and Applied Genetics 120, 985–995 20012263 10.1007/s00122-009-1226-8

[CIT0026] Umezawa T Nakashima K Miyakawa T Kuromori T Tanokura M Shinozaki K Yamaguchi-Shinozaki K 2010 Molecular basis of the core regulatory network in ABA responses: sensing, signaling and transport. Plant and Cell Physiology 51, 1821–1839 20980270 10.1093/pcp/pcq156 PMC2978318

[CIT0027] Wang Y Wu Y Duan C 2012 The expression profiling of the CsPYL, CsPP2C and CsSnRK2 gene families during fruit development and drought stress in cucumber. Journal of Plant Physiology 169, 1874–1882 22959675 10.1016/j.jplph.2012.07.017

[CIT0028] Wang Y Ying J Kuzma M 2005 Molecular tailoring of farnesylation for plant drought tolerance and yield protection. The Plant Journal 43, 413–424 16045476 10.1111/j.1365-313X.2005.02463.x

[CIT0029] Xu ZY Lee KH Dong T 2012 A vacuolar beta-glucosidase homolog that possesses glucose-conjugated abscisic acid hydrolyzing activity plays an important role in osmotic stress responses in *Arabidopsis* . The Plant Cell 24, 2184–2199 22582100 10.1105/tpc.112.095935 PMC3442595

