# Supplementary material for: Overexpression of PYL5 in rice enhances drought tolerance, inhibits growth, and modulates gene expression
Source: J Exp Bot. 2014 Jan 27;65(2):453–64. doi: 10.1093/jxb/ert397 (PMC3904710; doi:10.1093/jxb/ert397)
Supplement: Supplementary Data [file supp_65_2_453__index.html]

Overexpression of PYL5 in rice enhances drought tolerance, inhibits growth, and modulates gene expression — Supplementary Data 

# Overexpression of *PYL5* in rice enhances drought tolerance, inhibits growth, and modulates gene expression

## Supplementary Data

Data files

**Files in this Data Supplement:**

- Supplementary Data - Supplementary Data
