# Supplementary material for: Overexpression of PYL5 in rice enhances drought tolerance, inhibits growth, and modulates gene expression
Source: J Exp Bot. 2014 Jan 27;65(2):453–64. doi: 10.1093/jxb/ert397 (PMC3904710; doi:10.1093/jxb/ert397)
Supplement: Supplementary Data [file supp_ert397_jexbot105171_file001.pdf]

Over-expression of OsPYL5 in rice enhances drought tolerance, inhibits the growth and modulates gene expression

Hyunmi Kim, Kyeyoon Lee, Hyunsik Hwang, Nikita Bhatnagar, Dool-Yi Kim, In Sun Yoon, Myung-Ok Byun, Sun Tae Kim, Ki-hong Jung, Beom-Gi Kim

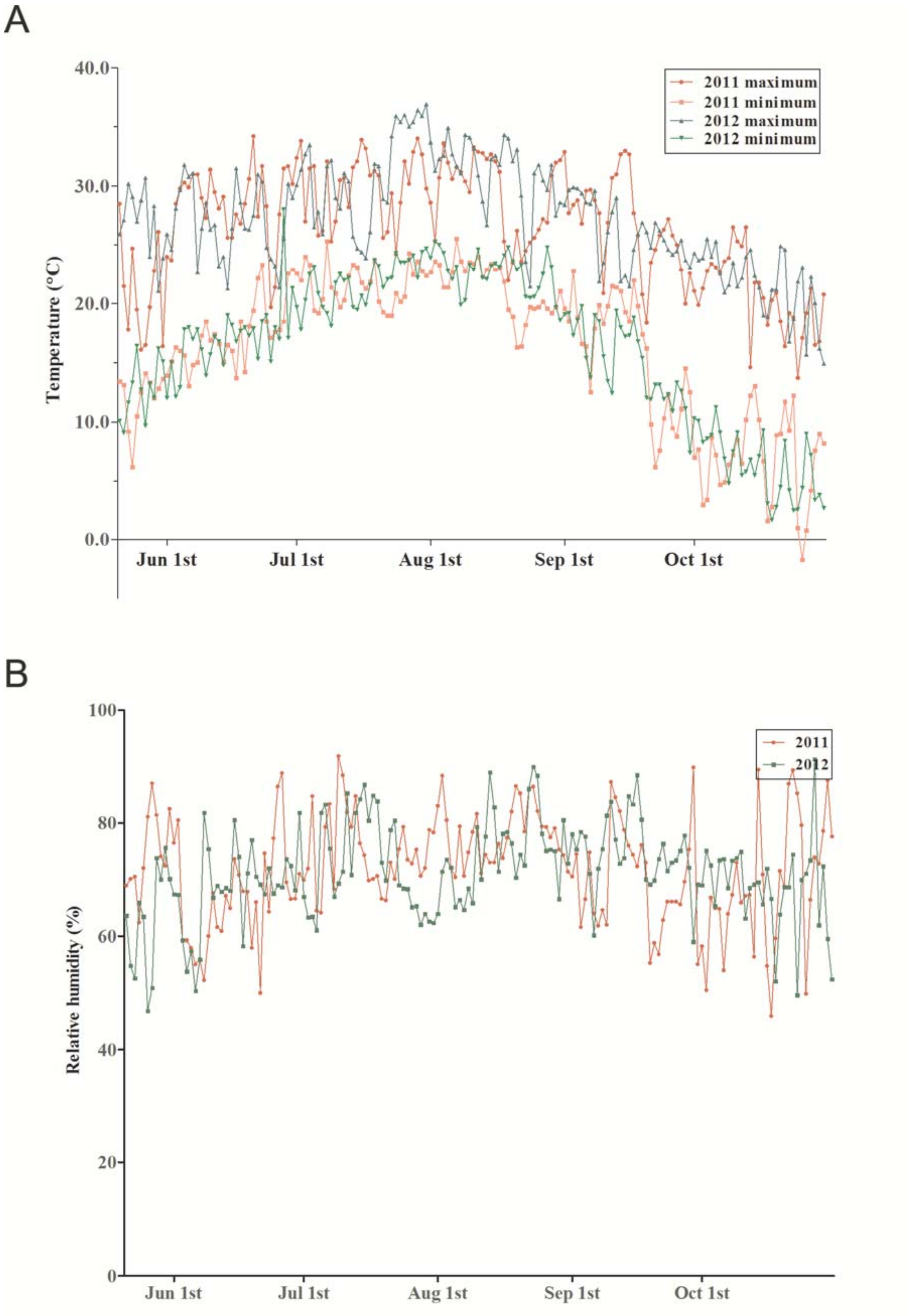

Figure S1. Seasonal variations of temperatures and relative humidities of the rice paddy field in the years 2011 to 2012
